# Comparative Genomic Analysis of COMT Family Genes in Three *Vitis* Species Reveals Evolutionary Relationships and Functional Divergence

**DOI:** 10.3390/plants14132079

**Published:** 2025-07-07

**Authors:** Yashi Liu, Zhiyuan Bian, Shan Jiang, Xiao Wang, Lin Jiao, Yun Shao, Chengmei Ma, Mingyu Chu

**Affiliations:** College of Horticulture, Gansu Agricultural University, Lanzhou 730070, China; 1073323120913@st.gsau.edu.cn (Y.L.);

**Keywords:** grapevine, three *Vitis* species, COMT gene family, abiotic stress, expression pattern

## Abstract

Caffeic acid-O-methyltransferase (COMT) is a key enzyme in lignin synthesis and secondary metabolism in plants, and it participates in the regulation of plant growth and development as well as plants’ stress response. To further investigate the function of COMT in grapevine, a total of 124 *COMT* family genes were identified from three *Vitis* species in this study, namely Pinot noir (*Vitis vinifera* L.), *Vitis amurensis*, and *Vitis riparia*. The amino acid sequence encoded by these genes ranged from 55 to 1422 aa, and their molecular mass ranged from 6640.82 to 77,034.43 Da. Subcellular localization prediction inferred that they were mainly located in the plasma membrane and cytoplasm. The prediction of secondary structures showed that α-helix and irregular coiled-coil were primary structural elements. These genes were unevenly distributed across 10 different chromosomes, respectively. Phylogenetic tree analysis of the amino acid sequences of VvCOMT, VaCOMT, VrCOMT, and AtCOMT proteins showed that they were closely related and were divided into four subgroups. The motif distribution was similar among the cluster genes, and the gene sequence was notably conserved. The 124 members of the COMT gene family possessed a variable number of exons, ranging from 2 to 13. The promoter region of all of these *COMTs* genes contained multiple cis-acting elements related to hormones (e.g., ABA, IAA, MeJA, GA, and SA), growth and development (e.g., endosperm, circadian, meristem, light response), and various stress responses (e.g., drought, low temperature, wounding, anaerobic, defense, and stress). The intraspecies collinearity analysis suggested that there were one pair, three pairs, and six pairs of collinear genes in *Va*, Pinot noir, and *Vr*, respectively, and that tandem duplication contributed more to the expansion of these gene family members. In addition, interspecific collinearity revealed that the *VvCOMTs* had the strongest homology with the *VaCOMTs,* followed by the *VrCOMTs*, and the weakest homology with the *AtCOMTs*. The expression patterns of different tissues and organs at different developmental stages indicated that the *VvCOMT* genes had obvious tissue expression specificity. The majority of *VvCOMT* genes were only expressed at higher levels in certain tissues. Furthermore, we screened 13 *VvCOMT* genes to conduct qRT-PCR verification according to the transcriptome data of *VvCOMTs* under abiotic stresses (NaCl, PEG, and cold). The results confirmed that these genes were involved in the responses to NaCl, PEG, and cold stress. This study lays a foundation for the exploration of the function of the COMT genes, and is of great importance for the genetic improvement of abiotic stress resistance in grapes.

## 1. Introduction

Lignin is an important phenolic polymer that can be found in the secondary cell walls of plants and plays a crucial role in maintaining plants’ mechanical strength, water transport, and growth and development [1]. Structurally, the composition of lignin monomers can be divided into three main types: (1) butylated lignans (S-lignans), which are derived from the polymerization of butylated propane structural monomers, (2) guaiacylated lignans (G-lignans), which are formed by the polymerization of guaiacylated propane structural monomers, and (3) guaiacylated lignans (G-Lignans), which are formed by the polymerization of p-hydroxyphenylpropane structural monomers [2]. Lignin is polymerized from p-hydroxyphenylpropane monomers to p-hydroxyphenyl lignin (p-hydroxyphenyl lignin, H-lignin) [3]. In gymnosperms, the lignin is predominantly guaiacyl lignin (G), dicotyledons contain predominantly guaiacyl-seryl lignin (G-S), and monocotyledons contain mostly guaiacyl-seryl-p-hydroxyphenyl lignin (G-S-H) [4]. The synthesis of lignin monomers can be divided into three pathways: the phenylpropane universal metabolic pathway, the lignin-specific synthetic pathway, and the glycosylated transport and polymerization process of lignin monomers [5]. Among them, caffeic acid O-methyltransferase (COMT) plays multiple roles in lignin synthesis. It catalyzes the methylation of caffeic acid to form ferulic acid, converts 5-hydroxyphenylaldehyde to sinapaldehyde, and also catalyzes the transfer of a methyl group from S-adenosyl-L-methionine (SAM) to form ferulic acid, which generates S-adenosyl-L-homocysteine (SAH) as a byproduct [6,7]. Therefore, COMT is considered a key enzyme in the biosynthesis of lignin monomers.

The cell wall serves as a barrier for plants against biotic and abiotic stresses, and caffeic acid O-methyltransferase (COMT) is involved in lignin synthesis. Therefore, COMT plays an important regulatory role in plants’ response to biotic and abiotic stresses. The expression of the maize *ZmCOMT* gene results in decreased lignin content, a reduced S/G ratio, and improved lignin digestibility [8], whereas, the *ZmCOMT1* knockout mutants exhibit reduced drought tolerance, increased leaf ROS (reactive oxygen species) accumulation, the exogenous application of the COMT substrate caffeic acid, restored drought tolerance [9]. Under low-temperature stress, the expression level of *TaCOMT-3* in wheat (*Triticum aestivum* L.) was upregulated, and *TaCOMT-3* methylated hydroxycinnamic acid to produce cinnamic acid, which enhanced the cell membrane’s stability and activated the CBF (C-repeat binding factor) pathway, and thereby improved the cold tolerance of wheat [10]. The overexpression of *PtCOMT* in poplar (*Populus* L.) led to an increase in the S/G (syringyl/guaiacyl) ratio of lignin monomers and improved its drought tolerance and cell wall mechanical strength [11]. In addition, COMT has been found to be involved in the regulation of melatonin biosynthesis in some plants. Yan et al. [12] found that overexpressing *SlCOMT1* increased the melatonin content in transgenic tomato leaves and significantly enhanced the salt and drought tolerance of transgenic tomato plants compared to the wild type. The melatonin content was elevated in transgenic *Arabidopsis thaliana* plants overexpressing *ClCOMT1*, and the plants’ tolerance to drought, low temperature, and salt stress was enhanced [13]. In Rice (*Oryza sativa* L.), *OsCOMT1* regulated the synthesis of lignin and melatonin by catalyzing the methylation of 5-hydroxyferulic acid and 5-hydroxypineal, thereby playing a role in the growth, development, and stress response of plants [14]. In summary, COMT can improve plants’ resistance to abiotic stress by regulating the synthesis and accumulation of lignin and melatonin.

Currently, *COMT* gene families have been reported in many plants, with 17 *COMT* genes having been identified in *A. thaliana* [15], 21 in melon (*Cucumis melo*) [16], 33 in rice [14], 25 in camellia (*Camellia sinensis*) [17], 31 in maize(*Zea mays*) [18], 33 in walnut [19], and 16 in watermelon (*Citrullus lanatus*) [13]. However, the characterization and functional elucidation of the *COMT* gene family in grapevine remain largely unexplored. Therefore, we systematically identified *COMT* family members in Pinot noir (*Vitis vinifera* L.), *Vitis amurensis* (*Va*), and *Vitis riparia* (*Vr*) using their genomic information, and conducted protein physicochemical property analyses, chromosomal localization, cis-acting element analyses, conserved motif analyses, phylogenetic tree analysis, covariance analysis, codon preference analysis, and evolutionary pressure selection. Meanwhile, we analyzed the expression profiles of *VvCOMT* genes in different tissues and organs at various growth and developmental stages, as well as their expression patterns under different abiotic stresses, using transcriptome data from NCBI. Finally, we validated the relative expression levels of selected *VvCOMT* members under drought, low-temperature, and salt stress using qRT-PCR, providing a theoretical basis for further exploring the function of grape *COMT* genes and innovating germplasm resources.

## 2. Results

### 2.1. Identification of COMT Genes in the Grapevine Genome and Analysis of Their Physicochemical Properties

In this study, we identified the members of the grape *COMT* gene family using the hidden Markov model (HMM) combined with BlastP search. A total of 61 *VaCOMT*, 45 *VvCOMT*, and 36 *VrCOMT* genes were identified in *Va*, Pinot noir, and *Vr*, respectively. These genes were named based on their chromosomal positions, namely, *VaCOMT1*–*VaCOMT54*, *VvCOMT1*–*VvCOMT36*, and *VrCOMT1*–*VrCOMT34* (Table S1). The physicochemical properties of these genes were analyzed using the Expasy online software (https://web.expasy.org/protparam/), which showed that the lengths of the VaCOMT amino acids ranged from 108 to 848 aa, that their relative molecular weights ranged from 11,542.45 to 94,273.35 Da, that their isoelectric points ranged from 4.81 to 9.27, and that their protein instability index ranged from 28.67 to 45.52. Among them, 9 VaCOMT members were stable proteins (protein instability index < 40), while the remaining 45 members of the VaCOMT group were unstable proteins (protein instability index > 40). For the VvCOMTs in Pinot noir, the amino acid length ranged from 55 to 1422 aa, the relative molecular weight from 6640.82 to 1,622,260.96 Da, the isoelectric point from 4.84 to 9.21, and the protein instability index from 26.9 to 46.21. Regarding the VrCOMTs in *Vr*, the amino acid length ranged from 251 to 669 aa, the relative molecular mass from 27,469.82 to 77,034.43 Da, the isoelectric point from 5.29 to 8.61, and the protein instability index from 28.42 to 49.51. Among the VrCOMT members, 5 were stable proteins, and the remaining 29 members were unstable proteins. The predicted subcellular localization results indicated that the COMT family members in three *Vitis* species were primarily localized in the plasma membrane and cytoplasm.

The prediction results of the secondary structure indicate that the COMT proteins from the three *Vitis* species lack a β-turn and are primarily composed of α-helix, extension strands, and random coil. In the VaCOMTs, the proportion of the α-helix structure ranged from 25.11% to 55.5%, that of the random coil structure from 34.47% to 53.29%, and that of the extended strands structure from 9.92% to 18.72%. In the VvCOMTs, the α-helix ranged from 33.76% to 54.14%, the random coils from 34.08% to 46.92%, and the extended strands from 7.67% to 15.2%. In the VrCOMTs, the α-helix structure ranged from 35.43% to 51.69%, random coils ranged from 36.2% to 55.61%, and the extended strands ranged from 8.97% to 14.29%. Overall, the random coil structure constituted the largest proportion, while the extended strand structure constituted the smallest proportion of the secondary structure of these COMT proteins. This distribution pattern may represent a unique feature of the secondary structure of the COMT family in grapevine.

### 2.2. Phylogenetic Tree of COMT Gene Family in Different Species

**Figure 1:**
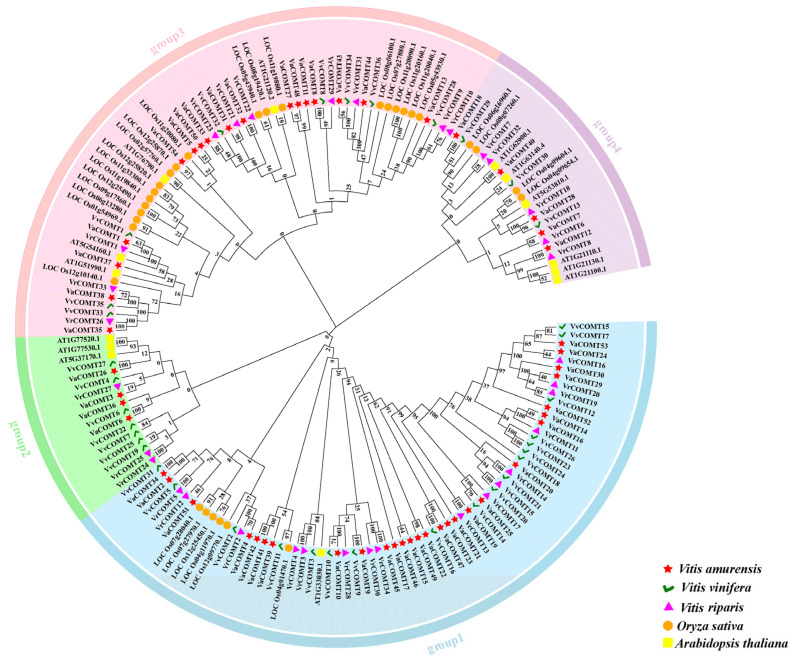
Phylogenetic trees of *COMT* family genes in different species. The five different colored shapes represent COMT proteins from four different species. The red triangle represents *Vitis amurensis*, the green sign represents *Vitis vinefera*, the purple triangle represents *Vitis riparis*, the orange circle represents *Oryza sativa*, and the yellow square represents *Arabidopsis thaliana*.

The protein sequences of 171 COMT genes from *Va*, Pinot noir, and *Vr*, rice, as well as *Arabidopsis*, were subjected to multi-sequence alignment using Clustal Muscle, and the phylogenetic tree was constructed using MEGA7.0 [20]. Based on the phylogenetic analysis of the COMTs, we classified them into four groups (Figure 1). Specifically, Group 1 was comprised of 69 members, including 25 VaCOMTs, 17 VvCOMTs, 16 VrCOMTs, 6 OsCOMTs, and 1 AtCOMTs. Group 2 included 17 members, consisting of 4 VaCOMTs, 7 VVCOMTs, 3 VRCOMTs, and 3 ATCOMTs. Group 3 consisted of 59 members, including 17 VaCOMTs, 8 VvCOMTs, 10 VrCOMTs, 20 OsCOMTs, and 4 AtCOMTs. There are 23 members in Group 4, which comprises 5 VaCOMTs, 3 VvCOMTs, 5 VrCOMTs, 4 OsCOMTs, and 6 AtCOMTs. Overall, the COMT genes of the three *Vitis* species are closely related to those of rice and *Arabidopsis*. It is implied that the *COMT* genes of these three species may share certain functional similarities with those of rice and *Arabidopsis*.

### 2.3. Chromosomal Localization of the Grape COMT Gene Family

**Figure 2:**
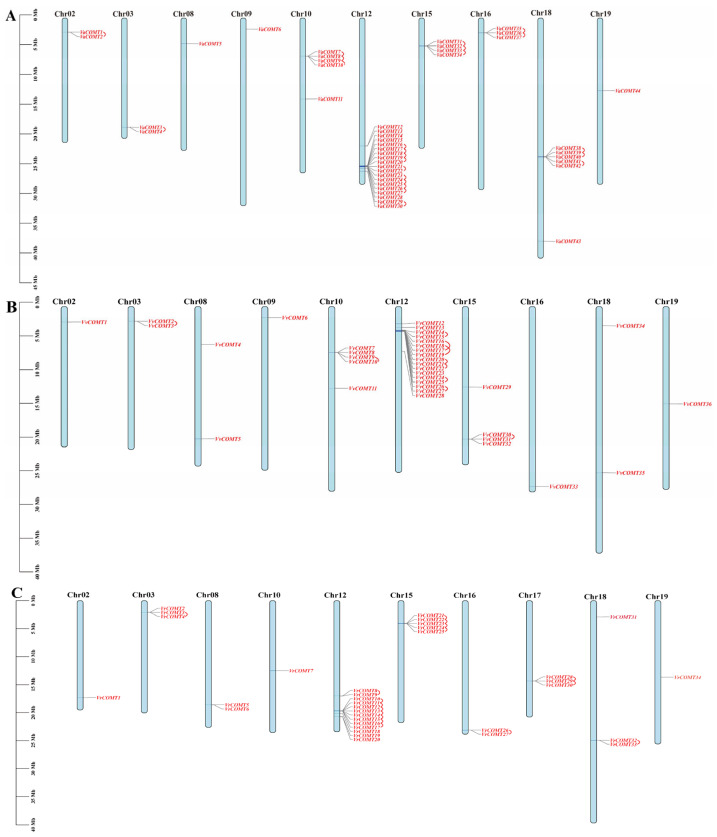
Localization of *COMT* genes on chromosomes in three *Vitis* species. Chromosome numbers are shown at the top of each bar. Gene names are shown in black. Scale bars are on the left. The red curve represents the tandem-duplication genes. (**A**) Chromosomal distribution of *VaCOMT*, (**B**) chromosomal distribution of *VvCOMT*, and (**C**) chromosomal distribution of *VrCOMT*.

The chromosomal distribution of the *COMT* gene families in three *Vitis* species was visualized using TBtools-II v2.313. In *Va*, 44 *COMT* genes were unevenly distributed across 10 chromosomes (Figure 2), namely Chr2, Chr3, Chr8, Chr10, Chr12, Chr15, Chr16, Chr18, Chr19, and ChrUn. Additionally, there were 10 genes located on various scaffolds. Specifically, only one gene was found on each of Chr8, Chr9, Chr19, and ChrUn. The chromosome with the highest number of genes (19) was Chr12, which accounted for 43.18% of the total genes (Figure 2A). Notably, 16 genes were clustered on Chr12. The gene pairs *VaCOMT1*/*VaCOMT2*, *VaCOMT3*/*VaCOMT4*, *VaCOMT7*/*VaCOMT8*, *VaCOMT9*/*VaCOMT10*, *VaCOMT31*/*VaCOMT32*, *VaCOMT35*/*VaCOMT36*, *VaCOMT36*/*VaCOMT37*, *VaCOMT38*/*VaCOMT39*, *VaCOMT39*/*VaCOMT40*, and *VaCOMT41*/*VaCOMT42* are located on Chr2, Chr3, Chr10, Chr15, Chr16, and Chr18, respectively. Based on their sequence alignment similarity (75%) and physical distance (<100 kb) [21], we speculate that the gene pairs *VaCOMT1*/*2*, *3*/*4*, *7*/*8*, *9*/*10*, *16*/*17*, *18*/*19*, *20*/*21*, *22*/*23*, *24*/*25*, *26*/*27*, *29*/*30*, *31*/*32*, *33*/*34*, *35*/*36*, *37*/*38*, *39*/*40*, and *41*/*42* are tandem repeat genes.

In Pinot noir, 36 *COMT* genes are unevenly distributed across 10 chromosomes, namely Chr2, Chr3, Chr8, Chr10, Chr12, Chr15, Chr16, Chr18, and Chr19. Specifically, only one gene is located on Chr2, Chr9, Chr16, and Chr19. The chromosome with the largest number of genes (17) is Chr12, which accounts for 47.22% of the total genes (Figure 2B). Notably, 17 genes are clustered on Chr12. The gene pairs *VvCOMT2*/*VvCOMT3*, *VvCOMT9*/*VvCOMT10*, *VvCOMT30*/*VvCOMT31* are located on Chr3, Chr10, and Chr15, respectively. Based on the similarity of the sequence alignment and physical distance, *VvCOMT2*/*3*, *9*/*10*, *14*/*15*, *16*/*17*, *18*/*19*, *24*/*25*, *27*/*28*, and *30*/*31* are tandem repeat genes.

In *Vr*, 34 *COMT* genes are unevenly distributed across 10 chromosomes, namely Chr3, Chr4, Chr8, Chr10, Chr12, Chr15, and Chr19. Specifically, only one gene is located on each of Chr3, Chr4, and Chr19. The highest number of genes (13) is located on Chr12, which accounts for 38.23% of the total genes (Figure 2C). Notably, 13 genes are clustered on Chr12. The gene pairs *VrCOMT3*/*VrCOMT4*, *VrCOMT21*/*VrCOMT22*, *VrCOMT22*/*VrCOMT23*, *VrCOMT23*/*VrCOMT24*, *VrCOMT24*/*VrCOMT25*, *VrCOMT26*/*VrCOMT27*, *VrCOMT28*/*VrCOMT29*, *VrCOMT29*/*VrCOMT30*, *VvCOMT32*/*VrCOMT33* are located on Chr3, Chr15, Chr16, Chr17, and Chr18, respectively. Based on the similarity of their sequence alignment and physical distance, *VrCOMT3*/*4*, *8*/*9*, *10*/*11*, *12*/*13*, *14*/*15*, *16*/*17*, *21*/*22*, *22*/*23*, *24*/*25*, *26*/*27*, *28*/*29*, and *29*/*30*, *32*/*32* are tandem repeat genes.

In conclusion, there are both similarities and differences in the chromosomal distribution of COMTs among the three grapes species. In addition to 10 *VaCOMT* members being distributed on 10 scaffolds, the remaining *VaCOMT* genes were located on chromosomes 2, 3, 8, 9, 10, 12, 15, 16, 18, and 19, which were the same as in the distribution of *VvCOMT*s. However, for the *VrCOMT*s, there were three *VrCOMT* genes distributed on chromosome 17, but no genes on chromosome 9, while the rest of the *VrCOMT* genes were distributed on the same chromosomes as in the *VaCOMT* and *VvCOMT* genes. Moreover, the tandem duplication events in the three *Vitis* species occurred more frequently on Chr12 and exhibited a clustered distribution. Members of the *VaCOMT* and *VrCOMT* gene families are clustered on Chr15, Chr16, and Chr18, whereas the *VvCOMT* genes are primarily concentrated on chromosomes Chr3, Chr10, and Chr15.

### 2.4. Analysis of the Structure and Conserved Motif of the COMT Gene in Grapes

**Figure 3:**
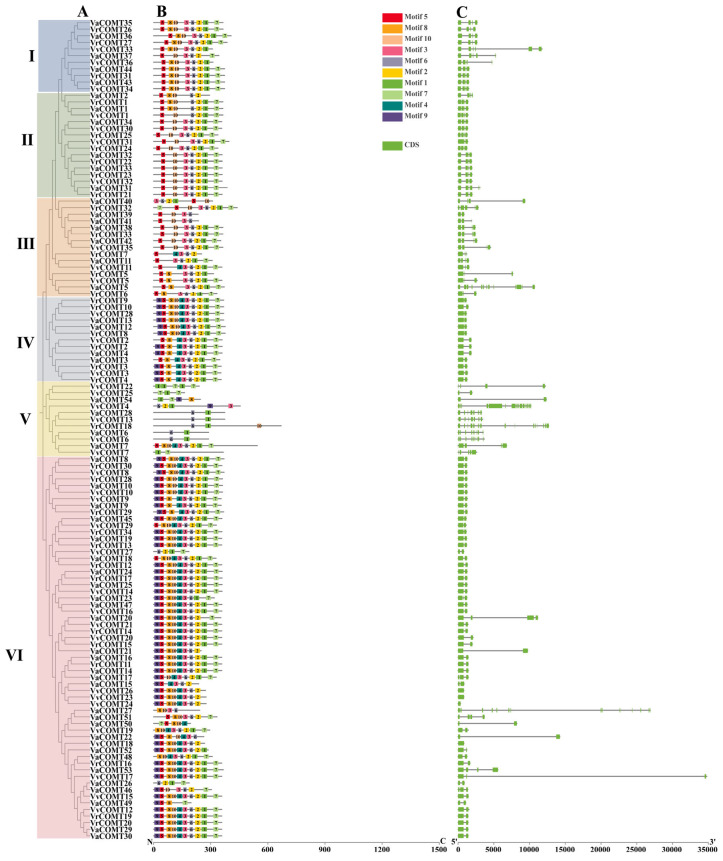
The phylogenetic relationship, conserved motif distribution and gene structures of *VaCOMTs*, *VvCOMTs*, and *VrCOMTs*. (**A**) The phylogenetic tree was constructed based on COMT protein sequences using MEGA 7.0 software and the NJ method. Bootstrap was repeated 1000 times [20], and detailed information of clustering is shown in different colors. (**B**) Motif composition of *COMT* members. (**C**) Exon-intron structure of *COMT*s, yellow boxes indicate untranslated regions 5 and 3; green boxes indicate exons. I, II, III, IV, V and VI represent the subgroup of COMT genes in three *Vitis*.

The phylogenetic analysis of 124 COMT members in three *Vitis* species was conducted, and the COMTs were classified into six groups (Figure 3A,B). The Group I members shared motif 5, motif 10, motif 3, and motif 6, with 10 members additionally containing motif 8, motif 2, and motif 1. However, VaCOMT37 lacks motif 8 and motif 1. The Group II members universally possessed motif 5, motif 6, motif 10, and motif 2, while 12 members also contained motif 3, motif 1, and motif 7. Notably, *VaCOMT2*, *VrCOMT1*, and *VaCOMT1* additionally included motif 8. The Group III members all contained motif 5, motif 3, and motif 6, with five members also harboring motif 10, motif 2, motif 1, and motif 7. The Group IV members consistently shared motif 5, motif 8, motif 4, motif 3, motif 6, motif 2, motif 1, and motif 7, with six members additionally possessing motif 9 and motif 4. Group V included 11 members containing motif 1. VvCOMT22, VvCOMT25, VvCOMT54, VaCOMT7 and VvCOMT7 uniquely contained motif 7, whereas VaCOMT28, VvCOMT13, VaCOMT6, VvCOMT6, and VrCOMT18 were distinguished by the presence of motif 6 and motif 10. The Group VI members exhibited the highest motif diversity, containing all 10 motifs (motif 9, motif 5, motif 8, motif 10, motif 4, motif 3, motif 6, motif 2, motif 1, and motif 7). Among them, 33 members displayed highly similar motif arrangements and compositions. In summary, the COMT protein sequences across the three *Vitis* species are relatively conserved, with minor variations in motif type, member, and distribution. While members within the same subgroup exhibit similar motif compositions, subtle differences exist between branches of the same subgroup.

The exon-intron structure of the *COMT* genes across the three *Vitis* species is presented in Figure 3C. The number of exons presented a considerable difference, ranging from 1 (*VaCOMT27*) to 16, and there were more exons within longer gene sequences. Interestingly, 70.3% of the *COMT* genes harbored either two or three exons, while four genes (*VvCOMT18*, *VvCOMT23*, *COMT24*, and *VvCOMT24*) had only one exon. The remaining 26 genes contained 5–16 exons. However, a few *COMT* genes exhibited significant variations in length due to alterations in intron length and quantity. For instance, *VvCOMT4* possessed the longest gene sequence, containing the highest number of exons (16).

### 2.5. Cis-Acting Element Analysis of VaCOMT, VvCOMT and VrCOMT Genes

**Figure 4:**
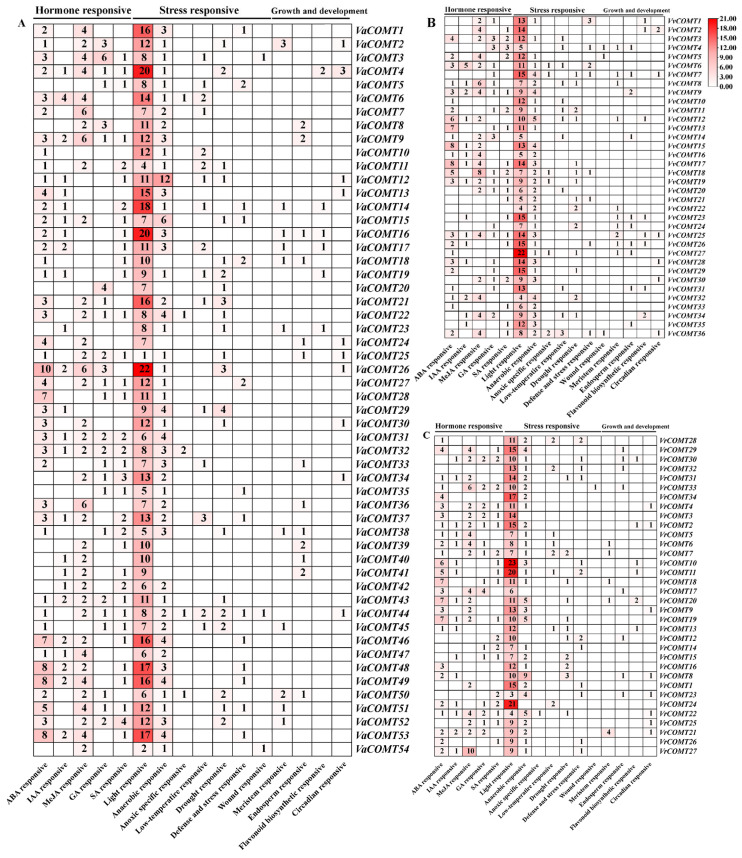
Cis-acting elements analysis of *COMT* genes in three *Vitis* species. The different colors and numbers in the grid indicate the number of different cis-acting elements in *VaCOMT*, *VvCOMT*, and *VrCOMT* genes. (**A**) *VaCOMT*, (**B**) *VvCOMT*; (**C**) *VrCOMT*.

To investigate the potential biological functions of the *COMT* genes in three *Vitis* species, we analyzed the cis-regulatory elements of 2000 bp upstream sequence from the translation start site (ATG) using the PlantCARE database (http://bioinformatics.psb.ugent.be/webtools/plantcare/html/). Our analysis revealed that all *COMT* promoters contained numerous cis-acting elements (Figure 4), which we categorized into three function groups; (1) plant hormone response, (2) stress response, and (3) plant growth and development regulation. Notably, all *COMTs* contained a large proportion of light response elements in their promoters, which implies that *COMTs* may play an important part in light-dependent signaling pathways.

Plant hormone-responsive elements, including ABA, MeJA, SA, GA, and IAA, were identified in the promoters of *COMT* genes, with each gene containing at least two distinct hormone-responsive elements. In *Va*, 43 *VaCOMT* gene promoters contained ABA-responsive elements, with the promoter of *VaCOMT36* containing the highest number of elements (10), which suggests that *VaCOMT36* is regulated by the ABA signaling pathway. In Pinot noir and *Vr*, ABA-responsive elements were widely distributed in the promoters of 24 *VvCOMTs* and 26 *VrCOMT*s, respectively. Specifically, the promoter of *VvCOMT15* and *VvCOMT17* contained the most ABA responsive elements (eight elements), followed by that of *VrCOMT18* and *VrCOMT20*, which contained seven ABA-responsive elements. Auxin-responsive elements were widely present in the promoters of 25 *VaCOMTs*, 15 *VvCOMTs*, and 16 *VrCOMTs*. Gibberellin-response elements were distributed in the promoters of 25 *VaCOMTs*, 19 *VvCOMTs*, and 15 *VrCOMTs*. MeJA-responsive elements were found in the promoters of 37 *VaCOMTs*, 19 *VvCOMTs*, and 19 *VrCOMTs*, with *VaCOMT36* containing the highest number of MeJA-responsive elements (six elements). Furthermore, the promoters of *VaCOMT4*, *VaCOMT9*, *VaCOMT4*, *VaCOMT31*, *VaCOMT43*, *VvCOMT9*, *VvCOMT19*, *VvCOMT25*, *VrCOMT2*, and *VrCOMT22* all contained responsive elements for ABA, MeJA, SA, GA, and IAA. The promoters of 17 *VaCOMTs*, 5 *VvCOMTs*, and 9 *VrCOMTs* contained four different types of hormone-responsive elements.

The promoters of 17 *VaCOMTs*, 5 *VvCOMTs*, and 9 *VrCOMTs*, respectively, contained four different kinds of hormone response elements. The promoters of 14 *VaCOMTs*, 13*VvCOMTs*, and 9 *VrCOMT*s, respectively, contained three different types of hormone response elements. These results demonstrated that hormone-responsive elements were most abundant in the promoter region of *VaCOMTs*, followed by *VvCOMTs* and *VrCOMTs*.

The stress response elements mainly included low temperature, drought, anaerobic induction, and defense and stress response elements. All promoter sequences of the *COMT* genes in three *Vitis* species contained at least one element related to the stress response. We also found that nearly all *COMT* genes contained anaerobic stress response elements. Specifically, hypoxia-specific inducible elements were identified in five *VaCOMTs* genes*,* six *VvCOMTs*, and one *VrCOMT* (*VrCOMT22*). Furthermore, low-temperature response elements, drought response elements, and defense and stress response elements were found in 52, 33, and 28 *COMT* genes, respectively, across the three *Vitis* species. Overall, promoter analysis suggested that *VaCOMTs*, *VvCOMTs*, and *VrCOMTs* might be involved in growth and development, phytohormone regulation, and the stress response. Moreover, the same cis-elements were observed among different promoter of *COMT* genes, both within and between the *Vitis* species.

### 2.6. Analysis of Duplication, Ka/Ks, and Codon Usage Bias of COMT Genes in Grape Genome

**Figure 5:**
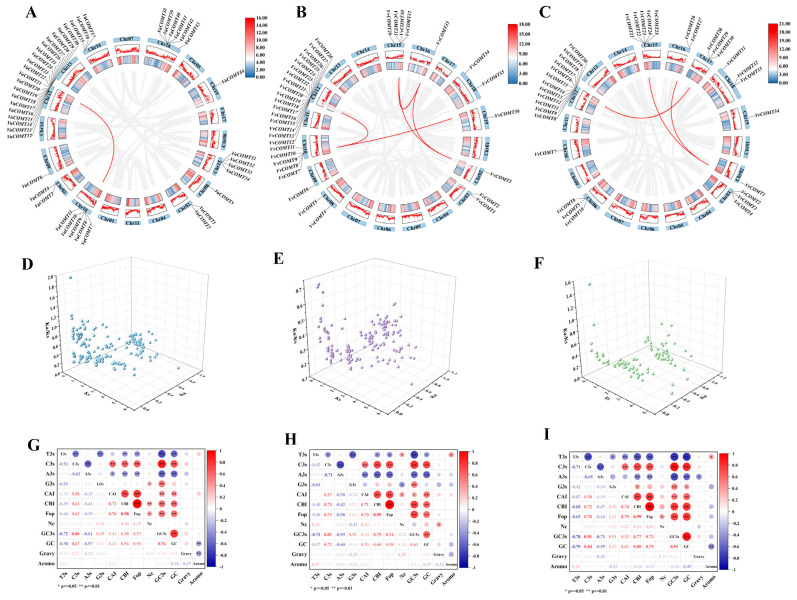
Synteny of interchromosomal relationships, Ka/Ks, and codon usage preference analysis of COMT genes in three *Vitis* species. The collinear relationship of *COMT* in *Va*, Pinot noir, and *Vr*, respectively. The blue bodies represent chromosomes, the gray lines represent all the collinear genes in grapes, and the red lines represent the collinear genes *VaCOMT*, *VvCOMT*, and *VrCOMT*. The number of chromosomes is indicated at the bottom of each chromosome (**A**–**C**). Blue represents the Ka/Ks of *VaCOMT* (**D**), purple represents the Ka/Ks of *VvCOMT* (**E**), and green represents the Ka/Ks of *VrCOMT* (**F**). Analysis of synonymous codon preferences and correlations of *VaCOMT*, *VvCOMT*, and *VrCOMT* genes: red indicates positive correlation, blue indicates negative correlation, and white indicates no correlation, significant correlations are represented with asterisks (* *p* < 0.05, ** *p* < 0.01). The U, C, A, and T of the third codon locus (U3s, C3s, A3s, G3s, and T3s), codon adaptation index (CAI), codon preference index (CBI), and optimal codon usage frequency (FOP). The number of valid codons (NC), the GC content of the third bit of the synonymous codon (GC3s) (**G**–**I**).

To further explore the evolutionary relationships of the *COMT* gene family in three *Vitis* species, intraspecific collinearity analysis was performed using one-step MCScanX in TBtools-II v2.313 (Figure 5). In *Va*, only one collinear gene pair was detected, namely *VaCOMT7*/*VaCOMT20*, which was located on Chr10 and Chr12, respectively (Figure 5A). In Pinot noir, six pairs of collinear *VvCOMT* genes were identified, including *VvCOMT8*/*VvCOMT17*, *VvCOMT9*/*VvCOMT14*, *VvCOMT7*/*VvCOMT36*, *VvCOMT32*/*VvCOMT33*, *VvCOMT30*/*VvCOMT1*, and *VvCOMT38*/*VvCOMT33*, which were located on Chr10/12, Chr10/12, Chr10/19, Chr15/16, Chr16/02, and Chr16/Chr19, respectively (Figure 5B). In *Vr*, three pairs of collinear *VrCOMT* genes were observed, including *VrCOMT1*/*VrCOMT21*, *VrCOMT1*/*VrCOMT26*, and *VrCOMT15*/*VrCOMT28*, which were located on Chr4/Chr15, Chr4/Chr16, and Chr12/Chr17, respectively (Figure 5C). Interestingly, the *COMT* genes in the Pinot noir genome exhibited the highest degree of collinearity. Furthermore, we speculated that these collinear genes, with their high sequence similarity, may share a common ancestral origin.

Ka and Ks represent non-synonymous substitution and the synonymous mutations rate, respectively [22]. The Ka/Ks is employed to measure the ration of non-synonyms to synonyms mutation, providing insights into selective pressures that act on genes during evolution [23]. In this study, we calculated the Ka/Ks ratio for 376 *VaCOMT*, 318 *VvCOMT*, and 194 *VrCOMT* genes pairs across three *Vitis* species. In *Va*, 368 gene pairs (97.9%) exhibited a Ka/Ks < 1, while 8 pairs (2.1%) showed a Ka/Ks > 1 (Figure 5D). Similarly, in *V. vinifera* cv. Pinot noir (Vv), all gene pairs displayed a Ka/Ks < 1 (Figure 5E). In *Vr*, 192 gene pairs (99.0%) had a Ka/Ks < 1, with only 2 pairs (1.0%) exceeding this threshold (Figure 5F).

These results suggest that the *COMT* gene family in three *Vitis* species has predominantly undergone strong purifying selection, with only a few genes expressing positive selection. This indicates that *COMT* genes likely maintain conserved biological functions following gene family expansion, although they may still be subject to regulation by environmental factors.

Codon usage preference contributes to further elucidating the genetic and evolutionary characteristic of COMT genes in grapevine. The relative synonymous codon usage frequency of three *Vitis* species was analyzed (Figure 5G–I). In *Va*, the effective number of codons (NC) of the *VaCOMT* genes ranged from 48.61 (*VaCOMT37*) to 61 (*VaCOMT49*), and the codon adaptation index (CAI) values ranged from 0.163 (*VaCOMT28*) to 0.239 (*VaCOMT39*). In Pinot noir, the NC values of the *VvCOMT* genes were in the range of 41.1 (*VvCOMT25*) to 60.65 (*VvCOMT30*), and the CAI values ranged from 0.163 (*VvCOMT13*) to 0.229 (*VvCOMT1*). In *Vr*, the NC values ranged from 50.88 (*VrCOMT27*) to 61 (*VrCOMT25*), and the CAI values ranged from 0.17 (*VrCOMT30*) to 0.235 (*VrCOMT33*). We analyzed the RSCU values of *VvCOMT*, *VrCOMT*, and *VaCOMT* proteins. As shown in Table S3, the number of COMT proteins that preferentially used codons with RSCU values >1 was higher in *VaCOMT* than in the *VvCOMT* and *VrCOMT* proteins.

Furthermore, correlation analysis of codon usage parameters revelated that T3s was positively correlated with C3s, G3s, G3s, CBI, and FOPs, while C3s was negatively correlated with CBI, FOPs, GC, and Gc3 (Figure 5G–I). However, no significant difference in codon usage parameters was observed among the three *Vitis* species.

**Figure 6:**
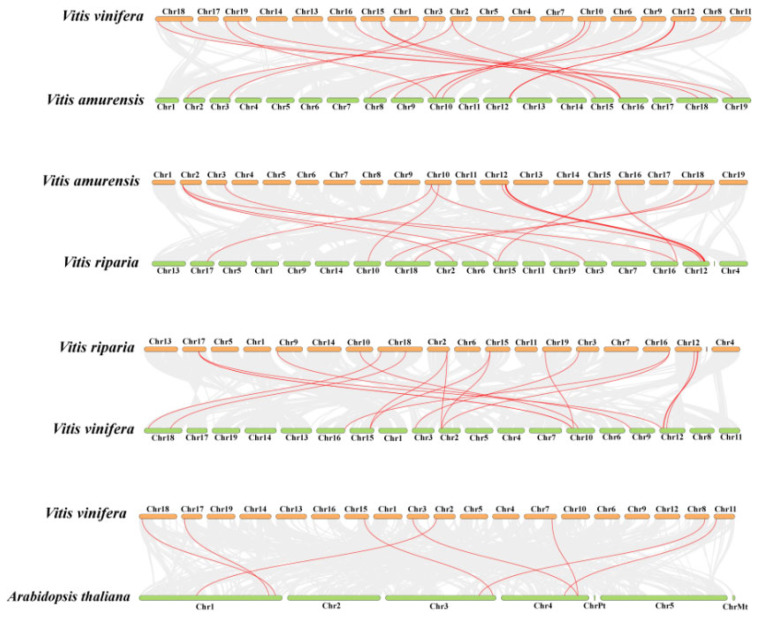
Collinearity of *VvCOMT* genes in four plant species. Gray lines in the background indicate homologous regions within the genomes of Pinot noir, *Vr*, *Va* and *Arabidopsis thaliana*; red lines highlight the collinearity of *COMT* gene pairs.

To further infer the evolutionary mechanisms of the COMT genes in three *Vitis* species, interspecific collinearity analyses were conducted between Pinot noir and *Va*, *Va* and *Vr*, *Vr* and Pinot noir, and Pinot noir and *Arabidopsis* (Figure 6). A total of 20, 17, 18, and 8 pairs of homologous genes were detected, respectively. The *VvCOMT* genes in Pinot noir exhibited the most collinear gene pairs with *VaCOMT* in *Va*, while the fewest collinear gene pairs were observed between Pinot noir and *Arabidopsis*. These results indicate that the *COMT* genes in the three *Vitis* species are highly conserved.

### 2.7. Expression Analysis of the Grape COMT Gene Family

#### 2.7.1. Expression Analysis of *VvCOMT* in Different Tissues

**Figure 7:**
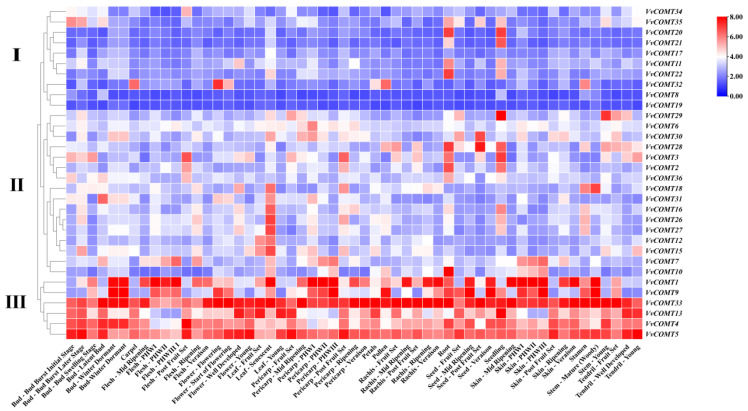
Expression pattern of *VvCOMT*s in different tissues at different growth and development stages. Transcriptome data were obtained from NCBI (GSE36128). Heatmap visualization was performed using TBtools-II v2.313. I, II and III represents the different expression patterns.

The expression profile of *VvCOMT* genes in different tissue and organs at various development stages (including roots, leaves, flowers, fruits, seeds, etc.) were obtained utilizing transcriptome data from NCBI (GSE36128). The expression of the *VvCOMTs* exhibited distinct tissue specificity (Figure 7). In general, the *VvCOMT* genes were divided into three groups according to their expression patterns. Group I included 10 genes, and their expression levels were low in a majority of tissues and organs, except *VvCOMT32*, which had a higher expression level in flowers, pollen, carpel, and stamens, and *VvCOMT20*, *VvCOMT21*, *VvCOMT11*, and *VvCOMT12*, which were expressed with a higher level in roots and seedlings. Group II includes *VvCOMT18*, *VvCOMT31*, *VvCOMT16*, *VvCOMT26*, *VvCOMT27*, *VvCOMT12*, and *VvCOMT15*, which are only expressed in specific tissues, such as senescent leaves. Group III included *VvCOMT33*, *VvCOMT13*, *VvCOMT4*, and *VvCOMT5.* These genes were highly expressed in various tissues and organs across different growth and development stages. Interestingly, *VvCOMT33* was highly expressed in tendrils, leaves, pericarp, flowers, rachis, skin, seeds, stem, tendril, and buds at different development stages. Additionally, *VvCOMT28*, *VvCOMT3*, *VvCOMT2*, and *VvCOMT10* exhibited higher expression levels in roots.

#### 2.7.2. Expression Analysis of *VvCOMT* Under Abiotic Stresses

**Figure 8:**
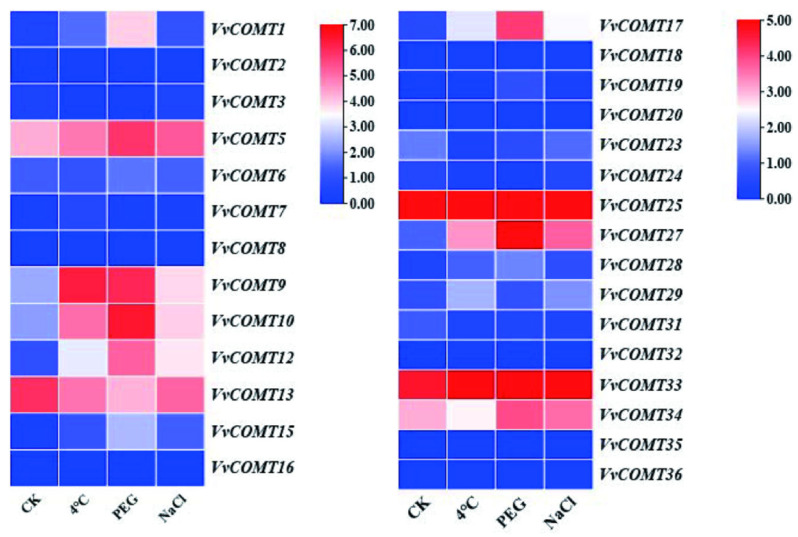
Expression pattern analysis of *VvCOMT*s under low temperature, salt, and drought stress. Transcriptome data were obtained from NCBI GEO database (GSE276430). The raw FPKM data were normalized as Log2 (FPKM + 1). Heatmap visualization was performed using TBtools-II v2.313.

The expression levels of *VvCOMT* genes under NaCl, PEG, and low-temperature stress were analyzed using transcriptome data from NCBI (GSE276430) (Figure 8). The results indicated that the expression levels of *VvCOMT5*, *VvCOMT9*, *VvCOMT10*, *VvCOMT13*, *VvCOMT26*, and *VvCOMT33* were significantly increased under cold (4 °C), PEG, and NaCl stress. The relative expression levels of some *VvCOMT* genes, such as *VvCOMT12* and *VvCOMT34*, were increased under PEG and NaCl stress. *VvCOMT1* and *VvCOMT17* exhibited significantly increase under PEG stress. Conversely, some genes were suppressed by one or more stresses, such as *VvCOMT3*, *VvCOMT6*, *VvCOMT7*, *VvCOMT28*, *VvCOMT29*, and *VvCOMT31*, which showed significantly decreased expression levels under cold (4 °C), PEG, and NaCl stress. Additionally, the relative expression levels of *VvCOMT34* and *VvCOMT12* were significantly decreased under cold stress (4 °C), while those of *VvCOMT1*, *VvCOMT15*, and *VvCOMT17* were significantly decreased under both cold (4 °C) and NaCl stress.

#### 2.7.3. qRT-PCR Analysis of VvCOMT Expression Patterns Under Abiotic Stresses

**Figure 9:**
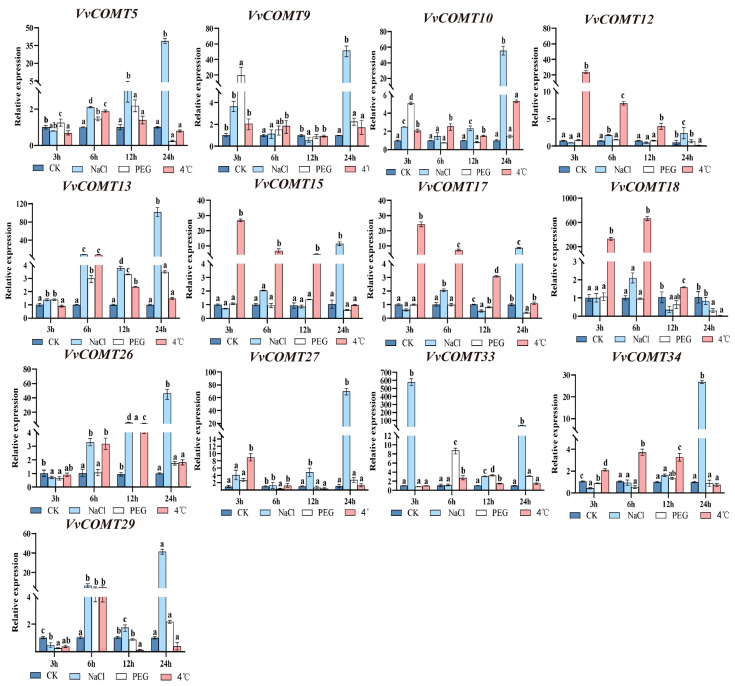
Expression patterns of *VvCOMT*s under NaCl, cold (4 °C), and PEG treatments. The expression levels of *VvCOMT* genes were normalized with elongation factor-1α (EF-1α) and calculated using the 2^−∆∆Ct^ method. The lowercase letters represent statistical significance at *p* < 0.05, one-way ANOVA.

Based on the transcriptome data of the *VvCOMT* genes under abiotic stresses such as NaCl, PEG, and cold (4 °C) stress, 13 *VvCOMT* genes were screened for qRT-PCR verification (Figure 9). The results showed that different *VvCOMT* genes exhibited distinct expression patterns under various abiotic stress. Under NaCl treatment, most of the genes were significantly upregulated at all points and peaked at 24 h, whereas *VvCOMT18* was downregulated at 12 and 24 h. Among the upregulated genes, the expression of *VvCOMT12* did not change significantly. Moreover, the relative expression level of *VvCOMT13* was more than 100-fold larger than control at 24 h.

Under cold stress (4 °C), most genes exhibited weak expression at all points, except for *VvCOMT12*, *VvCOMT13*, *VvCOMT15*, *VvCOMT17*, and *VvCOMT18*. Among these genes, the expression levels of *VvCOMT12*, *VvCOMT15*, and *VvCOMT17* were significantly upregulated at 3 h, 6 h, and 12 h, respectively, with the highest expression being observed at 3 h. The expression of *VvCOMT18* initially increased and then decreased, reaching a peak that was more than 660-fold higher than that of the control. These results indicated that these significantly expressed candidate genes played important roles in the response to NaCl, PEG, and low-temperature stress. Furthermore, the expression patterns of the *VvCOMT* genes were generally consistent with the presence of cis-elements in their promoters.

## 3. Discussion

COMT is a key enzyme in the plant phenylpropanoid pathway, participating in diverse biosynthetic pathways and secondary metabolic processes, such as the methylation modification of flavonoids, alkaloids, and stilbenes. It plays an important role in plant stress resistance and environmental adaptation [24]. The number of *COMT* gene family members varies among different plants. For example, 17 *AtCOMTs* and 33 *OsCOMTs* have been identified in the annual herbaceous model plants *A. thaliana* and rice, respectively. In this study, 54 *VaCOMTs*, 36 *VvCOMTs*, and 34 *VrCOMTs* were identified from *Va*, Pinot noir, and *Vr*, respectively. Compared with *A. thaliana* (17) and rice (33), the number of *COMT* genes in grapes is significantly higher, which indicates that this gene family has undergone substantial expression during the evolution of grapevine. Gene duplication, mainly including tandem duplication and segmental duplication, plays a significant role in biological evolution [25]. Interestingly, the number of tandem duplication gene pairs identified was much greater than that of collinear gene pairs found through intraspecific collinearity analysis in *Va*, Pinot noir, and *Vr*, respectively. These findings signify that tandem and segmental duplication are the primary drivers of this gene family’s expansion, with tandem duplication playing the dominant role.

The enzyme caffeic acid O-methyltransferase (COMT) is central to lignin biosynthesis [26]. Structure–function analyses of a caffeic acid O-methyltransferase from perennial ryegrass revealed the molecular basis for substrate preference. In this study, eight pairs of homologous genes were identified among 124 COMT genes in three *Vitis* species. Among these, *VvCOMT13* exhibited the highest expression levels in transcriptome data analysis under low temperature, salt, and drought stress. Based on these findings, we speculate that *VrCOMT18*/*VaCOMT28* may also exhibit high expression levels under similar stresses. The number of genes derived from the same ancestor gene varies among the three *Vitis* species. This inconsistency suggests that the three grape species may belong to distinct populations. Differences in origin and growth environment may have driven the expression of the *COMT* gene family to adapt to environmental pressures. Therefore, we hypothesize that tandem repetition has been a primary mechanism in the COMT gene family within the *Vitis* species. Similar findings have been reported during the expansion of this gene family in rice [27].

Although all 124 *COMT* genes in the three *Vitis* species possessed the caffeic acid-O-methyl transferase (COMT) conserved domain (PF00891), their basic characteristics, including coding sequence length, amino acid length, molecular weight, isoelectric point, and fat coefficient, were different. These results indicated that the COMT gene family has diverged among the different *Vitis* species during evolution. The secondary structure elements are predominantly composed of α-helices, extended strands, and random coils, with extended strands and random coils occupying a higher proportion. These findings are consistent with the characteristics of COMT secondary structure in maize [28], which suggests that this may represent a unique secondary structure feature of COMT proteins. Subcellular localization prediction indicates that COMT proteins in grape may be located in the plasma membrane, cytoplasm, and extracellular space. In a transient expression assay in tobacco, ClCOMT1-GFP genes were located in the cytoplasm [13]. This is similar to the results of our subcellular localization prediction, but there are differences, which indicates that these genes may perform different functions.

The COMT members from *A. thaliana*, rice, and three *Vitis* species were divided into four groups based on the phylogenetic tree. Group I, III, and IV each included members from three species, while group II only included member from rice and grapes Additionally, the protein similarity among *Va*, *Vr*, and Pinot Noi clustered as a distinct clade, was greater than 90%, such as VaCOMT10, VvCOMT10, and VrCOMT28. These findings indicated that the COMT genes in *Va*, Pinot noir, and *Vr* were highly conserved and exhibit a high degree of function similarity, with a lesser degree of differentiation during evolution. Transcriptome data analysis revealed that *VvCOMT10* was highly expressed under 4 °C and PEG stress. Similarly, we speculated that the expression of *VaCOMT10* and *VrCOMT28* may be induced by 4 °C and PEG stress. Furthermore, the cis-acting elements in the promoters of *VaCOMT10*, *VvCOMT10*, and *VrCOMT28* include ABA response elements and low-temperature response elements. Therefore, we hypothesize that *VaCOMT10*, *VvCOMT10*, and *VrCOMT28* may play an important role in low-temperature and drought resistance. Previous studies have shown that the rice *COMT* gene members *OsCOMT2*, *OsCOMT5*, *OsCOMT16*, and *OsCOMT21* are strongly induced under drought stress [14]. Therefore, members of the grape *COMT* gene family may also have similar functions.

In general, the distribution patterns (numbers and distribution position) of exons and conserved motifs for the COMT proteins in three *Vitis* species were highly similar to their corresponding phylogenetic grouping, suggesting that the *COMT* gene family is relatively conserved in grapevine. The number of exons of 124 *COMT* genes ranged from 1 to 16, with genes that had more exons generally exhibiting longer coding length sequences. This indicated that both the number and complexity of genes have increased as the number of exons has grown during the evolution. This finding is consistent with the relatively conservative sequence of the *COMT* gene in eucalyptus [29]. Addition, we also found that the type and number of cis-acting elements of *COMT* members within the same subgroup were also similar. The *COMT* genes clustered in the same branch of Group I and II all shared motif 5, which also contained ABA rand MeJA response elements. This indicated that strong conservation was observed between Group I and Group II, with similar cis-acting elements in their promoters, suggesting that they may play a similar functional role. Most COMT members of the three *Vitis* species located in the same branch of Group VI contained motif 1. Among the 18 *COMT* genes from three *Vitis* species, there are 18 low-temperature response elements and 19 drought response elements. The analysis of cis-acting elements suggests that these genes may be involved in the low-temperature and drought signal responses. Existing research has shown that genes located in the same subgroup of a phylogenetic tree also exhibit functional similarities due to their similar gene structures and conserved motifs [30].

The analysis of cis-acting elements revealed that the upstream promoter sequences of the *COMT* genes in three *Vitis* species primarily contain three categories of cis-acting elements, including hormone-responsive elements (ABA, IAA, MeJA, GA, and SA), growth and development elements, and defense stress response elements. These findings suggest that *COMT* members play important roles in growth and development, hormone signal transduction, and abiotic stress resistance. In citrus, *PtCOMT* has been shown to integrate ABA signals and regulate ABA synthesis to enhance root drought resistance [31]. The overexpression of *CICOMT1* in *A. thaliana* increased the melatonin content in transgenic plantlets and improved their resistance to abiotic stress [32]. Meanwhile, qRT-PCR results indicated that *VvCOMT15*/*17*/*18* containing more than 5 ABA response elements was significantly up-regulated under low-temperature and salt stress, which suggests that these genes may be involved in the ABA-dependent low-temperature signaling pathway and melatonin synthesis. These results are consistent with the previous research regarding CsCOMT1 in cucumber, which demonstrated that GATA3–COMT1–Melatonin acts as an upstream signaling of ABA, participating in selenium-enhanced cold tolerance by regulating the iron uptake and distribution in *Cucumis sativus* L. [32]. *VvCOMT9* and *VvCOMT10* exhibited similar sequence and expression patterns under NaCl and PEG treatment and were identified as tandem-duplication genes. This illustrates that tandemly repeated *COMT* genes in grape exhibited functional redundancy, and that their homologous genes (*VaCOMT35*/*VrCOMT26*) are likely to participate in salt and drought resistance. Moreover, *VvCOMT33* and *VvCOMT13* were highly expressed by under NaCl and PEG induction, which suggests that they play important roles in salt and drought resistance. Thereby, it can be inferred that the homologous genes of *VvCOMT33* (*VaCOMT35*/*VrCOMT18)* and *VvCOMT13* (*VaCOMT28*/*VrCOMT15)* likely have a similar function role in salt and drought stress. Under cold stress (4 °C), *VvCOMT12*/*15*/*17*/*18* exhibited the highest relative expression level at 3 h, which suggests that these genes play a particular role in low-temperature resistance. In brief, the *COMT* genes in grapes play an important role in abiotic stress resistance. However, in this study, only Pinot noir suspension cells under abiotic stress were used as material to analyze the expression level of *VvCOMTs*. Further in-depth research is required to investigate whether these genes show a similar expression pattern in grape plants, or whether their homologous genes in *Vr* and *Va* express similar functions.

## 4. Materials and Methods

### 4.1. Plant Materials and Methods

Grapevine suspension cells of ‘Pinot noir’ were cultured in GamborgB5 liquid medium supplemented with 0.1 mg L^−1^ naphthyl acetic acid, 0.2 mg L^−1^ kinetin, 200 mg L^−1^ casein hydrolysate, and 20 g L^−1^ sucrose [33] at pH 6.0 under darkness. After 7 days of culture, grape suspension cells were collected by funnel filtration. A total of 4 g of cells was weighed and added to 20 mL of B5 liquid medium. The drought and salt stress were induced by PEG (10%) and NaCl (200 mmol-L^−1^) treatments, respectively, and these were followed by incubation at 25 °C on a shaker in the dark. Low-temperature stress was induced at 4 °C. At four time points (3 h, 6 h, 12 h, and 24 h) after incubation, the cells were collected. Untreated suspension cells at each time point were used as the control. Three biological replicates were performed for each treatment. Cell samples were stored at −80 °C.

### 4.2. Identification of COMT Genes in the Grapevine Genome

The complete genome information of *Va* was downloaded from the VITSGDB database (http://vitisgdb.ynau.edu.cn/downloads.html, accessed on 20 July 2024) [34]. The genome information of *Vr* was obtained from NCBI database (https://www.ncbi.nlm.nih.gov/bioproject/PRJNA512170/, accessed on 10 December 2023) [35]. The genomic data of Pinot noir (*Vitis vinifera* L.) were downloaded from Telomere-to-Telomere assembly and annotation of Pinot Noir 40024 (https://zenodo.org/records/8245793, accessed on 18 July 2024) [36]. The sequence of *A. thaliana* AtCOMT was downloaded from Arabidopsis thaliana Information Resource (TAIR) database (https://www.arabidopsis.org/, accessed on 2 August 2024), and the sequence of *Oryza sativa* L. OsCOMT was obtained from the Ensemblplants database (https://plants.ensembl.org/Oryza_sativa/Info/Index, accessed on 2 August 2024).

Firstly, the hidden Markov model of COMT conserved domain (PF00891), downloaded from the Pfam database (http://pfam.xfam.org/, accessed on 6 August 2024), was employed to analysis the protein sequences using “hmmsearch” in HMMER3.1 software. Secondly, the homologous genes in three *Vitis* species were identified by applet BLAST (E value < 1 × 10^−5^) in TBtools-II v2.313 using the protein sequence of AtCOMT and OsCOMT as a query [37]. The candidate protein sequences obtained from the two methods were then verified using the Conserved Domain Database (CDD, https://www.ncbi.nlm.nih.gov/Structure/cdd/wrpsb.cgi, accessed on 8 August 2024) and SMART (http://Smart.embl-Heidelberg.de/, accessed on 10 August 2024) [38].

### 4.3. Physicochemical Properties and Localization of COMT Proteins

The basic physical and chemical properties such as the amino acid content, relative molecular weight, isoelectric point, hydrophilicity, and instability coefficient were analyzed using the online tool ExPASy (https://web.expasy.org/protparam/, accessed on 11 August 2024) [39]. CELLOv2.5 Subcellular localization predictor (http://cello.life.nctu.edu.tw/, accessed on 13 August 2024) was employed to predict the subcellular localization of COMTs [40]. The secondary structures were predicted by employing the SOPMA program (https://npsa-prabi.ibcp.fr/cgi-bin/npsa_automat.pl?page=/NPSA/npsa_sopma.html, accessed on 12 August 2024).

### 4.4. Phylogenetic Tree Construction of COMT Proteins in Different Species

ClustalW was used to perform multiple sequence alignment of COMT protein sequences from three *Vitis* species, *A. thaliana*, and rice, and then the phylogenetic tree was constructed via MEGA 7 [20] using the neighbor-joining (NJ) method with the J++ model and a bootstrapping value of 1000. Finally, the phylogenetic tree was modified in Evolview (https://evolgenius.info//evolview-v2/#login, accessed on 17 August 2024).

### 4.5. Gene Structure, Conserved Motifs, and Cis-Acting Element Analysis

The gene structure was extracted from GFF3 file and visualized by TBtools-II v2.313 [41], and the conserved motif of the COMT protein sequences in three *Vitis* species were predicted by employing the online tool MEME (MEME Suite 5.5.4), with the maximum number of motifs set to 10 and remaining parameters set to the default; the results were visualized using TBtools-II v2.313 software [42].

The genomic sequences 2000 bp upstream of the start codons of each *VaCOMT*, *VvCOMT*, and *VrCOMT* gene were extracted from GFF3 file of three *Vitis* species. Cis-acting elements were predicted using the online database PlantCARE (http://bioinformatics.psb.ugent.be/webtools/plantcare/html/, accessed on 14 August 2024), and visualized using TBtools-II v2.313 [42].

### 4.6. Chromosomal Localization and Synteny Analysis of COMT Genes

Chromosome localization of *COMT* genes in three *Vitis* species was achieved using their genome database resource and visualized employing TBtools-II v2.313 [42]. To analyze the collinearity among different *COMT* genes, the CDS. fasta file and transcripts.gff (or gff3) file of Arabidopsis were downloaded from the TIAR database. Interspecific collinearity and intraspecific collinearity of *COMT* genes pairs with other COMT genes were calculated and visualized using MCscanX program of TBtools-II v2.313 [42,43].

### 4.7. Codon Usage Bias and Ka/Ks Analysis

The CDS sequences of *VaCOMT*, *VvCOMT*, and *VrCOMT* genes were used to calculate COMT codon usage bias using CodonW1.4.2 software [44]. The codon usage preferences were based on a previous study [45]. This analysis covered multiple aspects, such as the RSCU evaluation, codon adaptation index (CAI), codon currency index (CBI), assessment of the frequency of optimal codons (FOPs), examination of GC3s and GCs, etc. The ratio of nonsynonymous (Ka) to synonymous (Ks) nucleotide substitutions (Ka/Ks ratio) was calculated using the Simple Ka/Ks Calculator 3.0 [46].

### 4.8. Gene Expression Data Source and Visualization

According to the homologous gene ID number, the FPKM values of *VvCOMT* genes in various tissues, including 54 tissues sampled at different growth and development periods, such as roots, stems, leaves, tendrils, floral organs, pulp, pericarp, and seeds, were obtained from the NCBI database GEO (Gene Expression Omnibus) (NCBI accession number; GSE36128) [47] and used to analyze the tissue-specific expression patterns of *VvCOMTs*. The expression patterns of *VvCOMT* genes were also analyzed utilizing publicly available transcriptomic data (GSE276430) at 0 h and 48 h after treatment with cold (4 °C), drought (PEG6000), and salt (NaCl) [48]. Finally, the raw FPKM data were normalized to Log2(FPKM+1), and the expression heatmap was presented using TBtools-II v2.313 [42].

### 4.9. RNA Extraction and Real-Time Quantitative PCR (qRT-PCR)

RNA was extracted from grapevine cells using the Trizol kit (Takara, Dalian, China). cDNA synthesis was performed using PrimeScript RT Reagent Kit with gDNA Eraser (Takara, Dalian, China). Specific primers for the *VvCOMT* genes were designed using Primer5.0 and synthesized by Shanghai Sangon Biotechnology Company (Shanghai, China). qRT-PCR was employed using SYBR^®^ Premix Ex Taq™ II (Takara, Dalian, China). These reactions were carried out under the following conditions: 95 °C for 30 s, 40 cycles at 95 °C for 15 s, 60 °C for 60 s. The relative expression level of genes was normalized with elongation factor-1 (*EF-1α*, VIT_206S0004g03240) and calculated by the 2^−ΔΔCt^ method [49]. Statistical analysis was conducted using SPSS version 26.0, while pairwise comparison was conducted by using the Tukey’s HSD method.

## 5. Conclusions

In this study, a total of 124 COMT gene family members were identified in three *Vitis* species—Pinot Noir, *Vr*, and *Va*, respectively—andthe physicochemical properties, cis-acting elements, gene structures, conserved motifs, chromosomal physical location, codon preferences, and intraspecific collinearity among *VrCOMT*s, *VvCOMT*s, and *VaCOMT*s exhibited certain differences. Interspecific collinearity analysis showed 20 pairs, 17 pairs, 18 pairs, and 8 pairs of homologous genes between *VvCOMTs* and *VaCOMTs*, *VaCOMTs* and *VrCOMTs*, *VrCOMTs* and *VvCOMTs*, and *VvCOMTs* and *AtCOMTs*, respectively. The Ka/Ks analysis indicated that COMT genes in three *Vitis* were subjected to purifying selection during evolution. Finally, under PEG, NaCl, and 4 °C treatment, different members presented distinct spatial and temporal expression patterns, which provides a basis for further research on the function of the COMT genes in resistance to abiotic stress in *Vitis* ssp. and lays the foundation for molecular breeding in the future.

## Data Availability

Data are contained within the article or Supplementary Materials.

## Supplementary Materials

The following supporting information can be downloaded at: https://www.mdpi.com/article/10.3390/plants14132079/s1, Table S1: Physical and chemical properties of *COMT* genes in three *Vitis*; Table S2: Oligo Sequence of *VvCOMT* genes; Table S3: RSCU values of COMT proteins in three *Vitis.*
